# Health Communication and Behavioural Practice towards Ending Hepatitis B Virus in Southwest Nigeria

**DOI:** 10.1155/2020/4969687

**Published:** 2020-12-23

**Authors:** Evaristus Adesina, Davies Adeloye, Hezekiah Falola, Babatunde Adeyeye, Darlynton Yartey, Tolulope Kayode-Adedeji

**Affiliations:** ^1^Department of Mass Communication, Covenant University, Ota, Nigeria; ^2^Centre for Global Health, Usher Institute, University of Edinburgh, Edinburgh, UK; ^3^Department of Business Management, Covenant University, Ota, Nigeria

## Abstract

Responding to the international call for strategic information to understand viral hepatitis, this study investigated the health communication practice on hepatitis B virus in Southwest Nigeria. Existing studies on HBV in Nigeria have primarily concentrated on health practitioners and their patients while neglecting detailed empirical data on semiurban and urban demographic information. This study examines health communication channels as predictors of knowledge, attitude, and behavioural practices with an emphasis on three Southwest states (Lagos, Oyo, and Ogun) in Nigeria that have the highest prevalence rate of HBV. Data were gathered through a survey from a total of 600 respondents of Southwest Nigeria randomly selected through the multistage sampling technique. The hypotheses were tested with the use of multiple regression. The result reveals that health communication channels for hepatitis B virus management had a significant influence on knowledge (*F* = 12.708, Df = 581, *P* < 0.05, Sig. at 0.000), attitude (*F* = 3.430, Df = 581, *P* < 0.05, Sig. at 0.000), and preventive practices (*F* = 11.075, Df = 581, *P* < 0.05, Sig. at 0.000) of residents of Southwest Nigeria, respectively. The study concludes that health communication channels such as the television, Internet, radio, newspaper, and health workers positively influence the behavioural practices of residents of Southwest Nigeria. The study recommends the development of a nationwide communication system on HBV targeted at putting an end to the disease in line with the 2030 global elimination objective of Sustainable Development Goal 3.

## 1. Introduction

The concept of communication is now largely recognised to be fundamental to effective healthcare. Poor communication has an adverse effect on both infectious and noninfectious diseases. Communication is, therefore, no longer seen as an add-on; on the contrary, it is recognised as being at the heart of patient care. As Kreps [1] noted, communication is fundamental in generating, collecting, and sharing health facts. It is an underlying human process that enables individual and collective adaptation to health risks at many different levels [1].

Central to the challenge and the task of ending viral hepatitis, therefore, is health communication, which is the theme of this study. Health communication attempts to cut down and eliminate the risk factors of lifestyle health behaviours. It is important to note that the need to understand health information behaviour is increasingly gaining the interest of communication discipline researchers. The National Cancer Institute (1989), Duffy and Jackson [2], and Bath [3] have all established the role of communication in all aspects of the health of humanity, especially as it relates to improving personal and public health. This is premised on the belief that communication, when applied effectively, can create awareness and engender relevant behavioural changes. An integral function of health communication is the influence of individuals and communities, for improved wellbeing. The Centre for Disease Control (CDC) describes health communication as the utilisation of communication approaches to enlighten and influence individual and community decisions for positive lifestyle-related health behaviours [4]. Health communication, therefore, aims at reducing and eliminating the risk factors of lifestyle-related health behaviours [5]. Achieving this health behavioural change, however, requires access to adequate health information.

Communication of vital messages to varied and specific audiences has proven to effectively influence the knowledge, attitude, and belief of people towards healthy behavioural choices [6, 7]. Such successes are evident in endemic diseases such as HIV/AIDS, malaria [8], and polio [9, 10]. Since health communication revolves around the development of the right strategies, understanding health-related issues, and increased knowledge [11], its adoption in the elimination of hepatitis B virus (HBV) is essential. HBV infection has been recognised as an infectious disease of global health importance [12–14].

Hepatitis B virus is widely referenced to as a silent killer consequent upon the nonawareness of carriers. Studies have also shown that persons could be infected up to 10 years without knowing [15, 16]. In the submission of Ott et al. [17], HBV records a high mortality rate, both from acute infection and chronic disease conditions, and is positioned among the top ten killer diseases globally. The World Health Organisation (WHO) has estimated that over 325 million people were living with HBV globally, with this accounting for 1.34 million yearly mortality rates from acute infection and hepatitis-related liver cancer and cirrhosis [12]. These estimates were similar to that of HIV and tuberculosis, estimated at 1.1 million deaths and 1.4 million deaths in 2016, respectively [12].

Popping et al. [18] have further revealed that 4.5 million premature deaths could be avoided in low- and middle-income countries by 2030. The government of Nigeria has turned towards addressing the burden of hepatitis B virus in the country more keenly. Hence, for the first time, on the 30th of July 2015, Nigeria joined the rest of the world in commemorating the World Hepatitis Day, four years after the official declaration by the United Nations General Assembly. The Federal Ministry of Health in Nigeria has noted that more than 22.6 million Nigerians are infected with hepatitis B viruses, with many being healthy carriers [19]. Of this, about 19 million and over 3.6 million Nigerians are estimated to be infected with HBV and HCV [19].

The necessity to examine the influence of health communication for hepatitis B virus management in Nigeria is vital at this point when the subject has taken the centre stage and has assumed an optimum position in the policies of international agencies and various developed and developing nations. In the assertion of Nutbeam [20], health communication is, therefore, an essential approach to managing public health issues. Increasing health literacy levels among the population to understand and apply information relating to health issues and achieving a substantial impact on health behaviours [7, 21–23] are critical functions of health communication.

While several studies have been done to examine the knowledge, attitude, and practice of people towards hepatitis, there is paucity of data on the extent to which health communication influences knowledge, attitude, and practice primarily in semiurban and urban population. This study, therefore, investigates health communication channels as predictors of knowledge, attitude, and behavioural practices with an emphasis on three Southwest states (Lagos, Oyo, and Ogun) in Nigeria that have the most prevalence rate of hepatitis B virus.

## 2. Method of Study

Data for this study were obtained from a survey conducted among 600 dwellers of three Southwest states (Lagos, Oyo, and Ogun) acknowledged to have the most prevalence rate of hepatitis B virus. Umego et al. [24], while alluding to Mbaawuaga et al. [25], revealed a 14% incidence rate of HBV in Lagos, while in Ogun and Oyo states, Anaedobe et al. [26] peg the rate of prevalence at 8.0% and 8.3%, respectively. For delimiting the population to a controllable size, the multistage sampling technique was utilised. The researchers at the first stage stratified the three states into three senatorial districts each (Oyo South, Oyo Central, and Oyo North), (Ogun East, Ogun Central, and Ogun West), and (Lagos Central, Lagos West, and Lagos East). Two senatorial districts were therefore selected from each state making a total of six with the aid of the lottery method of random sampling. Senatorial districts at the second stage were stratified into local government areas (LGAs), with two selection from each senatorial district. In all, twelve LGAs were selected in all across the states. At the third selection stage, two wards were chosen from each local government making a total of 24 wards. Using the lottery method, two streets were selected each from the ward resulting in forty-eight selected streets. In the fifth stage, the streets were stratified into residential houses; the researcher made use of the systematic sampling technique to select the residential houses that fall into the sample. The questionnaire employed for data collection was primarily designed by the researchers and distributed in line with what is already established in the literature precisely to elicit response on the variables under consideration.

For ethical consideration, all the participants were informed about the set objective of the study. They were allowed to discontinue their participation at any level without providing a reason(s) for the decision. Therefore, only those who are willing participated in the survey. Besides, the respondents stayed anonymous with a promise that all their responses will be treated with topmost confidentiality. Verbal consent was obtained from the respondents. The intent and background of the study were stated, and respondents were kept abreast of the process of participation.

Also, the validity of the research instrument was subjected to face and content validity, while Cronbach alpha was used for the reliability of the study instrument. Furthermore, a pilot study to establish the validity and reliability of the instrument was conducted. Sixty copies of the questionnaire, which represents 10% of the total number of respondents, were administered to respondents in the Ota, Ado-Odo Local Government Area of Ogun state. The 10% sample size for the pilot study was recommended by Baker (1994). The pilot result shows that data were normally distributed. Statistical Package for Social Science 23 was used for the coding of the data, while the regression analytical tool was used for the analyses of the tested hypotheses.

## 3. Results

Table 1 shows the result of the age distribution of the respondents across the three states selected (Lagos, Ogun, and Oyo). The result shows that of 582 respondents in the three selected states, the rate of those within the age bracket of below 18 is 6.9%. It is also important to note that of 582 respondents, 30.8% fall within the age bracket of 18–25. In the 25–35 age category, there was a total of 29.7% representation. Furthermore, the age categorisation of 36–45 has a 19.9% representation in the entire sample size of 582 respondents. The table further shows the cross tabulation of the respondents' gender according to the selected states. The table depicts that 57.4% were male and 42.6% were female. It was observed that male respondents were more than their female counterparts, but the margin is not wide. It is essential to state that majority of the respondents were students with a 25.3% representation.

H_o1_: health communication channels for hepatitis B virus management have no significant influence on the knowledge of residents in Southwest Nigeria.

In order to determine whether health communication channels for hepatitis B virus management have no significant influence on the knowledge of residents in Southwest Nigeria, a multiple regression analysis was computed.

The model summary of the analysis in Table 2 shows the level of the variance in the dependent variable (knowledge of residents) is explained by the independent variable (health communication channels). In this case, the *R* square is 0.197; if expressed by a percentage, it will be 19.7%. This suggests that 19.7% of the variance in health communication channels can be explained by the variance in the knowledge of residents. The adjusted *R* square shows 0.181, that is, 18.1% variability of the independent variable (radio, television, newspapers, leaflets, Internet, health workers, colleagues, neighbours, counsellors, relatives, and seminars), while the standard error of the estimate indicates 0.677, which signifies error term. This means that a unit increase in health communication channels (particularly Internet, colleagues, radio, and television) will lead to a rise in residents' knowledge on hepatitis B virus infection.

The ANOVA shows that the *F* value is 12.708 at 0.000 significance level. The implication is that, collectively, health communication channels have a significant influence on residents' knowledge on hepatitis B virus infection. It must, however, be noted that it is not all the communication channels that predict residents' knowledge, as depicted in Table 2. Therefore, health communication channels for HBV management have a significant influence on the knowledge of residents on HBV infection.

**Figure 1:**
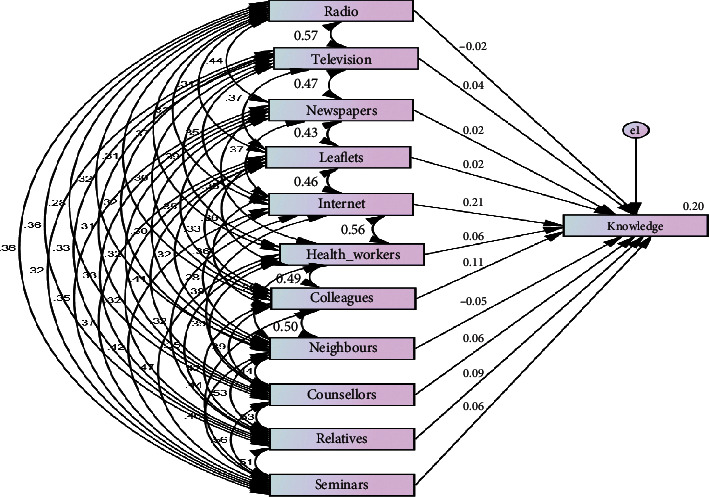
Health communication channels and residents' knowledge on the hepatitis B virus model.

The coefficient table, the structural equation modeling depicted in Figure 1, and the standard regression weights show the model that expresses the extent to which health communication channels influence residents' knowledge on hepatitis B virus.  Table 3 and Figure 1 also depict the model that shows the extent to which health communication channels influence residents' knowledge on hepatitis B virus management and which of the health communication channels contributed to the prediction of the residents' knowledge on HBV. The comparison of the contribution of various health communication channels is determined by beta value, as shown in the coefficient table (See Table 3).

Table 3 and Figure 1 reveal that the Internet as a means of health communication channel had more statistical significance in predicting residents' knowledge on hepatitis B virus. It recorded the highest beta value (beta = 0.212, significance 0.000) followed by colleagues (beta = 0.108, significance 0.025).

The significance level below 0.05 implies statistical confidence of above 95%. This means that the health communication channels have a significant influence on residents' knowledge on hepatitis B virus. Thus, the null hypothesis (H_01_) was rejected; therefore, health communication channels for HBV management have a significant influence on the knowledge of residents on hepatitis B virus infection.

H_o2_: health communication channels for hepatitis B virus management have no significant effect on attitude of residents in Southwest Nigeria.

The model summary of the analysis in Table 4 reveals the extent to which the variance in the dependent variable (attitudes of residents) is explained by the independent variable (health communication channels). In this case, the *R* square is 0.062; if expressed by a percentage, it will be 6.2%. This suggests that 6.2% of the variance in health communication channels can be explained by the variance in attitudes of residents towards hepatitis B virus infection. The adjusted *R* square shows 0.044, that is, 4.4% variability of the independent variable (radio, television, newspapers, leaflets, Internet, health workers, colleagues, neighbours, counsellors, relatives, and seminars), while the standard error of the estimate indicates 0.581, which signifies error term.

The ANOVA shows the *F* value of 3.430 at 0.000^b^ significance level. The *P* value of the health communication channels has a significant influence on residents' attitudes on hepatitis B virus infection. It becomes imperative to note that of all the communication channels, the Internet had a more positive significant influence on the attitudes of the respondents.

**Figure 2:**
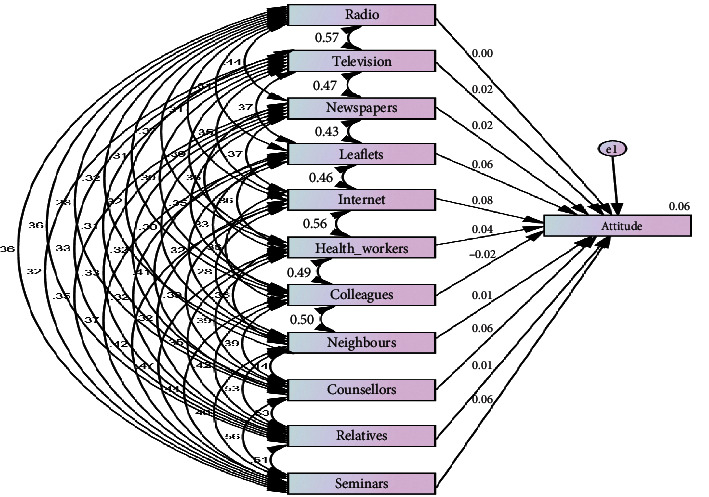
Health communication channels and residents' attitudes on the hepatitis B virus management model.

The coefficient Table 4, coupled with Figure 2,which shows the relationship between the model, and Table 5 that shows the standardised+ regression weights of the model expressed the extent to which health communication channels influence residents' attitudes on hepatitis B virus and which of the health communication channels contributed to the prediction of the residents' attitudes on HBV. The comparison of the contribution of various health communication channels is determined by beta value as shown in the coefficient table.

The model revealed that the Internet as a means of health communication channel had more statistical significance in predicting residents' attitudes on hepatitis B virus. It recorded the highest beta value (beta = 0.084, significance 0.011) followed by counsellors (beta = 0.061, significance 0.261). This means that, of all the health communication channels listed, the Internet makes the strongest contribution in explaining residents' attitudes on HBV than the other variables. The significance level below 0.01 implies statistical confidence of above 99%. This means that, collectively, health communication channels have significant influence on residents' attitudes towards HBV.

H_o3_: there is no significant influence of health communication channels for HBV management on preventive practices of residents in Southwest Nigeria.

The model summary of the analysis in Table 6 reveals the extent to which the variance in the dependent variable (preventive practices of residents) is explained by the independent variable (health communication channels). In this case, the *R* square is 0.176; if expressed by a percentage, it will be 17.6%. This suggests that 17.6% of the variance in health communication channels can be explained by the variance in preventive practices of residents against hepatitis B virus infection. The adjusted *R* square shows 0.160, that is, 16% variability of the independent variable (radio, television, newspapers, leaflets, Internet, health workers, colleagues, neighbours, counsellors, relatives, and seminars), while the standard error of the estimate indicates 0.686, which signifies error term.

The ANOVA shows the *F* value of 11.075 at 0.000 significance level. Sequel to the significant value, generally, health communication channels have a significant influence on preventive practices of residents against hepatitis B virus infection.

**Figure 3:**
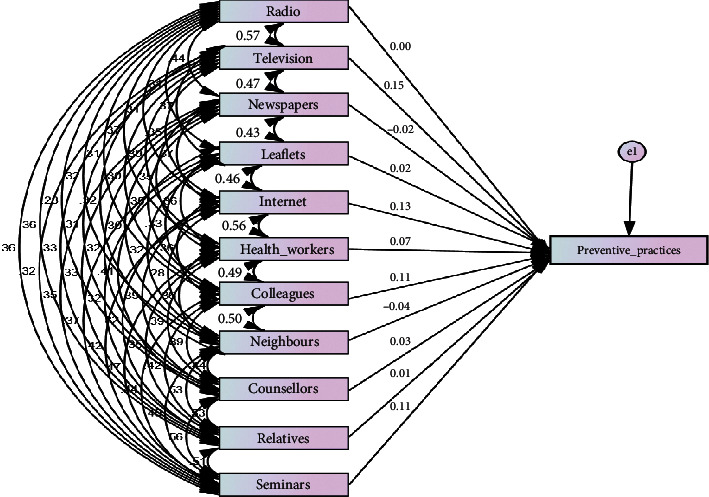
Health communication channels and residents' preventive practices against the HBV model.

The coefficients in Table 6, in addition to the results in Figure 3 which shows the relationship between the model, and Table 7 that shows the standardised regression weight of the model expressed the extent to which health communication channels influence preventive practices of residents against HBV infection. Table 7 also depicts the model that reveals the extent to which health communication channels influence preventive practices of residents against HBV infection and which of the health communication channels contributed to the prediction of the preventive practices. The comparison of the contribution of various health communication channels is determined by beta value, as shown in the coefficient table.

The model revealed that the television as a means of health communication channel had more statistical significance in predicting preventive practices of residents against HBV infection. It recorded the highest beta value (beta = 0.145, significance 0.003), followed by the Internet (beta = 0.129, significance 0.010). The significance level below 0.05 implies statistical confidence of above 95%. This implies that, collectively, health communication channels have a significant influence on preventive practices of residents against HBV infection. Thus, the null hypothesis (H_03_) was rejected; therefore, there is a significant influence of health communication channels for hepatitis B virus management on preventive practices of residents in Southwest Nigeria.

## 4. Discussion of Findings

Three hypotheses were tested with the use of multiple regression to determine the resultant effect of health communication strategies on knowledge, attitude, and preventive practice of residents of Southwest Nigeria. The first hypothesis tested if health communication channels for HBV management had no significant effect on the knowledge of residents of Southwest Nigeria. From the findings, the *F* value is 12.708 at 0.000^b^. Going by the *P* value of the standardised regression weights, the Internet has helped in providing effective information on hepatitis B virus. This means that of all the health communication channels listed, the Internet makes the most substantial contribution in explaining residents' knowledge on HBV than the other variables. The second hypothesis was stated in the null format that health communication channels for HBV management have no significant effect on the attitude of residents of Southwest Nigeria. The *F* value shows 3.430 at 0.000^b^, thereby making the null hypothesis to be rejected. The study indicates that a unit increase in health communication channels will lead to an increase in residents' attitudes on HBV infection. Health communication channels for hepatitis B virus management have a significant influence on the attitudes of residents on hepatitis B virus infection.

The last hypothesis tested if health communication channels for hepatitis B virus have no significance on the preventive practices of residents of Southwest Nigeria. The finding shows the *F* value of 11.075 at 0.000. Out of all, the communication channels in the prediction of respondents' preventive attitudes, only television, Internet, colleagues, and seminars have a significant influence on the preventive practices of the respondents.

The coefficient table, structural equation modelling, and the standard regression weight further revealed that the Internet as a means of communication contributed most to the high knowledge level and the attitude of residents of Southwest Nigeria. The changing pattern in disease burdens from infectious diseases to chronic diseases can be said to have influenced the desires of individuals to be knowledgeable and engage in behavioural practices towards HBV. Online health information can improve an individual's knowledge of, competence with, and engagement in health decision-making strategies.

Globally, the Internet has been acknowledged as playing a pivotal role in the health information system [27–33]. This medium of communication is said to be effective in the dissemination of health messages to diverse audiences, in the same manner the traditional media does. The submission of Michael and Cheuvront [27] alludes to this. In their view, the Internet can economically and geographically reach a mass population. Social media outlets such as Facebook, Instagram, Twitter, WhatsApp, and Snapchat have engendered this, as Nigeria's Ministry of Communication has stated that 75% of online users in Nigeria are utilising different social media platforms [34–37]. Also, the Nigeria Communication Commission, in its quarterly report, revealed that the country's Internet subscription rate reached 196,379,542 in June 2020 [38].

Studies have also revealed the efficacy of television, radio, newspaper, and magazine in promoting health communication in different diseases, as well as influencing behavioural changes [14, 39–41]. One cannot, however, conclude that the traditional media have not been useful in the dissemination of health information to the mass audience. It could be a situation of what Oyero [42] and Okorie et al [43] describes as media convergence, that is, the coming together of traditional media of communication on the Internet platform. The Internet, in the view of Oyero [42], “has eroded the differences among communication channels, thus unifying them up into one.” Indeed, we are in an era where all traditional media of communication now converge to reach a mass majority of the audiences. Several prints and broadcast media channels have their presence on various Internet channels such as websites, YouTube, Facebook, and Instagram, where people get real-time updates and information.

## 5. Conclusion

Achieving SDG 3 of combating hepatitis B virus cannot be done in isolation without the effective usage of health communication strategies. This study has established that the majority of Southwest residents use the information source of the Internet in seeking knowledge of behavioural practice information on hepatitis B virus. It can, therefore, be established that Internet technology is fast taking hold among the population segment in Nigeria. This result of this study aligns with the submission of the Statistic Bureau Agency in Nigeria, which has noted that Southwest states (Oyo, Lagos, and Ogun) are the most Internet-subscribed states in Nigeria as compared to other regions of the country. Despite an increase in health communication practice, in the region, there is, however, a need for the enunciation of a nationwide communication policy on HBV directed at putting an end to this scourge. In addition, there is a need for health regulatory bodies like Nigeria Centre for Disease Control, Federal Ministry of Health, and Non-Government Organisation working on HBV to leverage on the use of the Internet to further improve knowledge.

## Figures and Tables

**Table 1:** Demographic characteristics of participants.

		State			Total (*n* = 582) %
		Lagos state (*n* = 289) %	Ogun state (*n* = 119) %	Oyo state (*n* = 174) %	
Age	Below 18	2.1	2.4	2.4	6.9
	18–25	16.3	8.2	6.2	30.8
	26–35	15.5	4.1	10.1	29.7
	36–45	10.1	3.1	6.7	19.9
	46–55	4.5	2.1	3.6	10.1
	56–above	1.2	0.5	0.9	2.6
Total		49.7	20.4	29.9	100.0 *n* = 582

Gender	Male	29.9	11.3	16.2	57.4
	Female	19.8	9.1	13.7	42.6
Total		49.7	20.4	29.9	100.0 *n* = 582

Occupation	Trader	13.1	3.6	7.6	24.2
	Artisan	5.3	2.1	1.9	9.3
	White collar job	7.0	2.6	6.2	15.8
	Student	12.0	7.7	5.5	25.3
	Unemployed	4.5	1.0	2.1	7.6
	Others	7.7	3.4	6.7	17.9
Total		49.7	20.4	29.9	100.0 *n* = 582

**Table 2:** Coefficients of health communication channels and knowledge on hepatitis B virus.

Model		Unstandardized coefficients		Standardized coefficients		Sig.
		*B*	Std. error	Beta
1	(Constant)	2.263	0.095	23.880	0.000
	Radio	−0.010	0.028	−0.018	−0.368	0.713
	Television programmes	0.024	0.029	0.042	0.846	0.398
	Newspapers	0.013	0.028	0.022	0.468	0.640
	Leaflets/pamphlet/brochure/flyer/catalogue	0.013	0.027	0.021	0.458	0.647
	Internet/website	0.116	0.027	0.212	4.302	0.000
	Health worker(s)	0.034	0.028	0.062	1.217	0.224
	Colleagues	0.063	0.028	0.108	2.242	0.025
	Neighbour(s)	−0.034	0.030	−0.055	−1.129	0.259
	Counsellors	0.038	0.031	0.061	1.214	0.225
	Relatives	0.051	0.030	0.087	1.716	0.087
	Seminars/workshop/conferences	0.035	0.029	0.060	1.182	0.238
	*R* value: 0.444^a^					
	*R* ^2^ value: 0.197					
	*P* value: 0.000^b^					
	*F* value: 12.708					

Dependent variable: knowledge of residents.

**Table 3:** Standardized regression weights of health communication channels and knowledge on hepatitis B virus.

			Estimate	SE	CR	*P*
Knowledge	<---	Radio	−0.018	0.028	−0.371	0.710
Knowledge	<---	Seminars	0.060	0.029	1.193	0.233
Knowledge	<---	Television	0.042	0.028	0.854	0.393
Knowledge	<---	Relatives	0.087	0.030	1.732	0.083
Knowledge	<---	Newspapers	0.022	0.027	0.472	0.637
Knowledge	<---	Counsellors	0.061	0.031	1.226	0.220
Knowledge	<---	Leaflets	0.021	0.027	0.463	0.644
Knowledge	<---	Neighbours	−0.055	0.030	−1.140	0.254
Knowledge	<---	Internet	0.212	0.027	4.343	^*∗∗∗*^
Knowledge	<---	Colleagues	0.108	0.028	2.264	0.024
Knowledge	<---	Health workers	0.062	0.028	1.229	0.219

**Table 4:** Health communication channels and attitude of residents on hepatitis B virus.

Model		Unstandardized coefficients		Standardized coefficients	*T*	Sig.
		*B*	Std. error	Beta		
1	(Constant)	2.531	0.081		31.138	0.000
	Radio	0.000	0.024	−0.001	−0.018	0.986
	Television programmes	0.010	0.024	0.022	0.414	0.679
	Newspapers	0.011	0.024	0.023	0.464	0.643
	Leaflets/pamphlet	0.028	0.024	0.060	1.207	0.228
	Internet/website	0.037	0.023	0.084	1.577	0.015
	Health worker(s)	0.017	0.024	0.039	0.708	0.479
	Colleagues	−0.009	0.024	−0.018	−0.352	0.725
	Neighbour(s)	0.004	0.026	0.007	0.140	0.889
	Counsellors	0.030	0.027	0.061	1.126	0.261
	Relatives	0.005	0.026	0.010	0.185	0.854
	Seminars/workshop	0.026	0.025	0.058	1.054	0.292
	*R* value: 0.249^a^					
	*R* ^2^ value: 0.062					
	*P* value: 0.000^b^					
	*F* value: 3.430					

Dependent variable: attitudes of residents.

**Table 5:** Standardised regression weights of health communication channels and attitude of residents on hepatitis B virus.

			Estimate	SE	CR	*P*
Attitudes	<---	Radio	−0.001	0.024	−0.018	0.986
Attitudes	<---	Seminars	0.058	0.025	1.064	0.287
Attitudes	<---	Television	0.022	0.024	0.418	0.676
Attitudes	<---	Relatives	0.010	0.025	0.186	0.852
Attitudes	<---	Newspapers	0.023	0.024	0.468	0.639
Attitudes	<---	Counsellors	0.061	0.027	1.137	0.256
Attitudes	<---	Leaflets	0.060	0.023	1.219	0.223
Attitudes	<---	Neighbours	0.007	0.026	0.141	0.888
Attitudes	<---	Colleagues	−0.018	0.024	−0.355	0.723
Attitudes	<---	Internet	0.084	0.023	1.592	0.011
Attitudes	<---	Health workers	0.039	0.024	0.715	0.475

**Table 6:** Coefficients of health communication channels and preventive practices of HBV.

Model		Unstandardized coefficients		Standardized coefficients	*t*	Sig.
		*B*	Std. error	Beta		
1	(Constant)	2.051	0.096		21.356	0.000
	Radio	0.002	0.029	0.004	0.082	0.935
	Television	0.085	0.029	0.146	2.934	0.003
	Newspapers	−0.013	0.028	−0.021	−0.447	0.655
	Leaflets/pamphlet	0.010	0.028	0.016	0.347	0.729
	Internet/website	0.071	0.027	0.129	2.594	0.010
	Health worker(s)	0.040	0.029	0.072	1.390	0.165
	Colleagues	0.062	0.029	0.107	2.183	0.029
	Neighbour(s)	−0.024	0.031	−0.038	−0.777	0.437
	Counsellors	0.021	0.032	0.034	0.670	0.503
	Relatives	0.009	0.030	0.015	0.287	0.774
	Seminars/workshop	0.066	0.030	0.114	2.219	0.027
	*R* value: 0.420^a^					
	*R* ^2^ value: 0.176					
	*P* value: 0.000^b^					
	*F* value: 11.075					

Dependent variable: preventive practices of residents.

**Table 7:** Standardised regression weights of health communication channels and preventive practices of HBV.

			Estimate	SE	CR	*P*
Preventive practices	<---	Radio	0.004	0.028	0.083	0.934
Preventive practices	<---	Seminars	0.114	0.029	2.241	0.025
Preventive practices	<---	Television	0.146	0.029	2.962	0.003
Preventive practices	<---	Relatives	0.015	0.030	0.290	0.772
Preventive practices	<---	Newspapers	−0.021	0.028	−0.451	0.652
Preventive practices	<---	Counsellors	0.034	0.031	0.676	0.499
Preventive practices	<---	Leaflets	0.016	0.028	0.350	0.726
Preventive practices	<---	Neighbours	−0.038	0.030	−0.785	0.433
Preventive practices	<---	Internet	0.129	0.027	2.619	0.009
Preventive practices	<---	Colleagues	0.107	0.028	2.204	0.028
Preventive practices	<---	Health workers	0.072	0.028	1.403	0.161

## Data Availability

Data are available upon request to the corresponding author.

## References

[1] Kreps G. L. (2003). The impact of communication on cancer risk, incidence, morbidity, mortality, and quality of life. *Health Communication*.

[2] Duffy B. K., Jackson L. D. (1998). *Health Communication Research: A Guide to Developments and Directions*.

[3] Bath P. A. (2008). Health informatics: current issues and challenges. *Journal of Information Science*.

[4] Stockwell M. S., Fiks A. G. (2013). Utilizing health information technology to improve vaccine communication and coverage. *Human Vaccines & Immunotherapeutics*.

[5] Enwald H. (2013). *Tailoring Health Communication: The Perspective of Information Users’ Health Information Behaviour in Relation to their Physical Health Status*.

[6] Olusola B. A., Gometi E. A., Ogunsemowo O., Olaleye D. O., Odaibo G. N. (2017). High rate of Hepatitis B virus infection among hairdressers in Ibadan, Nigeria. *Journal of Immunoassay and Immunochemistry*.

[7] Adesina E., Oyero O., Okorie N. (2020). Assessment of health communication practice on hepatitis B in Southwest Nigeria. *Cogent Social Sciences*.

[8] Malaria R. B. (2014). *Malaria Behaviour Change Communication (BCC) Indicator Reference Guide*.

[9] Obregón R., Waisbord S. (2010). The complexity of social mobilization in health communication: top-down and bottom-up experiences in polio eradication. *Journal of Health Communication*.

[10] Goldstein S., MacDonald N. E., Guirguis S. (2015). Health communication and vaccine hesitancy. *Vaccine*.

[11] Muturi N. W. (2005). Communication for HIV/AIDS Prevention in Kenya: social-cultural considerations. *Journal of Health Communication*.

[12] WHO (2017). *Global Hepatitis Report, 2017*.

[13] WHO (2016). *Global Health Sector Strategy on Viral Hepatitis 2016–2021. Towards Ending Viral Hepatitis*.

[14] Evaristus A., Olusola O., Nelson O., Lanre A., Babatunde A., Darlynton Y. (2020). Data on information sources, knowledge and practice on Hepatitis b virus in Southwest Nigeria. *Data in Brief*.

[15] Seeff L. B. (1999). Natural history of hepatitis C. *The American Journal of Medicine*.

[16] Libbus M. K., Phillips L. M. (2009). Public health management of perinatal hepatitis B virus. *Public Health Nursing*.

[17] Ott J. J., Stevens G. A., Groeger J., Wiersma S. T. (2012). Global epidemiology of hepatitis B virus infection: new estimates of age-specific HBsAg seroprevalence and endemicity. *Vaccine*.

[18] Popping S., Bade D., Boucher C. (2019). The global campaign to eliminate HBV and HCV infection: international viral hepatitis elimination meeting and core indicators for development towards the 2030 elimination goals. *Journal of Virus Eradication*.

[19] Olawale G. (2016). *22.6 m Nigerians Suffering from Hepatitis*.

[20] Nutbeam D. (2008). The evolving concept of health literacy. *Social Science & Medicine*.

[21] Ishikawa H., Kiuchi T. (2010). Health literacy and health communication. *BioPsychoSocial Medicine*.

[22] Adesina E., Oyero O., Okorie N., Omojola O., Amodu L., Adeyeye B. Health management strategies for hepatitis care practices: an interplay of communication structures and social marketing theory.

[23] Adesina E., Oyero O., Okorie N., Ben-Enukora C., Adeyeye B. (2020). Risk communication for viral hepatitis management among migrants. *Handbook of Research on the Global Impact of Media on Migration Issues*.

[24] Umego C., Mboto C., Mbim E., Edet U., George U., Tarh J. (2018). Epidemiology of hepatitis B virus infection in South-South, Nigeria: a review. *International STD Research & Reviews*.

[25] Mbaawuaga E., Enenebeaku M., Okopi J. (2008). Hepatitis B virus (HBV) infection among pregnant women in Makurdi, Nigeria. *African Journal of Biomedical Research*.

[26] Anaedobe C. G., Fowotade A., Omoruyi C. E., Bakare R. A. (2015). Prevalence, socio-demographic features and risk factors of Hepatitis B virus infection among pregnant women in Southwestern Nigeria. *The Pan African Medical Journal*.

[27] Michael M., Cheuvront C. (1998). Health communication on the Internet: an effective channel for health behavior change?. *Journal of Health Communication*.

[28] Percheski C., Hargittai E. (2011). Health information-seeking in the digital age. *Journal of American College Health*.

[29] Jacobs W., Amuta A. O., Jeon K. C. (2017). Health information seeking in the digital age: an analysis of health information seeking behavior among US adults. *Cogent Social Sciences*.

[30] Amodu L., Omojola O., Okorie N., Adeyeye B., Adesina E. (2019). Potentials of internet of things for effective public relations activities: are professionals ready?. *Cogent Business & Management*.

[31] Usaini S., Nelson O., Bamgboye O., Amodu L., Afolabi F., Evaristus A. (2018). Internet, social media and computer-mediated relationship among engineering undergraduate students. *International Journal of Civil Engineering and Technology*.

[32] Adeyeye B., Amodu L., Odiboh O. (2019). Data on new media use for agricultural training and research at agricultural services and training centre (ASTC). *Data in Brief*.

[33] Okorie N., Oyedepo T., Amodu L., Adesina E., Afolabi F. (2019). Adopting indigenous languages in teaching communication and engineering education in tertiary institutions: lessons from South African Universities. *International Journal of Mechanical Engineering and Technology (IJMET)*.

[34] Amaefule E. (2017). *75% of Nigeria’a Online Population Use Social Media*.

[35] Omojola O., Amodu L., Okorie N., Imhonopi D., Yartey D., Adesina E. (2018). Assessing the one-lecture-one-test learning model in undergraduate journalism program using cohort design. *The Journal of Social Sciences Research*.

[36] Adesina E., Odiboh O., Oyero O., Adeyeye B., Yartey D., Ekanem T. Publishing African communication researches in open access outlets: an interrogation of scopus between 1996–2016.

[37] Odiboh O., Olonode A., Adesina E., Yartey D. Influence of e-communication and digital culture on Nigeria;s indigenous socio-cultural systems: a focus on Abeokuta and Ota, Nigeria.

[38] NCC (2020). *Internet Subscriber Data*.

[39] AbuSabha R. (2015). *Effective Nutrition Education for Behavior Change*.

[40] Mebane F. (2003). Examining the content of health care reporting: neither the health care system or policies creating it receive coverage they deserve. *Nieman Reports*.

[41] Stuart T. H., Achterberg C. (1997). *Education and Communication Strategies for Different Groups and Settings*.

[42] Oyero O. S. (2017). The implications of internet on the media and the practice of mass communication. *International Journal of Communication*.

[43] Okorie N., Amodu L., Jegede A., Adesina E., Martins O. (2019). Global media, digital journalism and the question of terrorism: an empirical inquest on ISIS. *Media Watch*.

